# MrHex1 is Required for Woronin Body Formation, Fungal Development and Virulence in *Metarhizium robertsii*

**DOI:** 10.3390/jof6030172

**Published:** 2020-09-14

**Authors:** Guirong Tang, Yanfang Shang, Shiqing Li, Chengshu Wang

**Affiliations:** 1 CAS Key Laboratory of Insect Developmental and Evolutionary Biology, CAS Center for Excellence in Molecular Plant Sciences, Shanghai Institute of Plant Physiology and Ecology, Chinese Academy of Sciences, Shanghai 200032, China; grtang@sippe.ac.cn (G.T.); yfshang@sibs.ac.cn (Y.S.); lishq1@shanghaitech.edu.cn (S.L.); 2 School of Life Science and Technology, ShanghaiTech University, Shanghai 201210, China; 3 CAS Center for Excellence in Biotic Interactions, University of Chinese Academy of Sciences, Beijing 100049, China

**Keywords:** Woronin body, conidiation, stress response, appressorium formation, virulence, *Metarhizium robertsii*

## Abstract

The Woronin body (WB) is a peroxisome-derived dense-core vesicle, a self-assembling hexagonal crystal of a single protein Hex1. This organelle is specific to the ascomycete fungi belonging to the Pezizomycotina subphylum by functioning in sealing septal pores in response to mycelium damage and the control of cell heterogeneity. We retrieved all available Hex1-domain containing proteins of different fungi from the GenBank database and found considerable length variations among 460 obtained Hex1 proteins. However, a highly conserved Hex1 domain containing 75 amino acid residues with a specific S/A-R/S-L consensus motif for targeting peroxisome is present at the carboxy-terminus of each protein. A homologous *Hex1* gene, named *MrHex1*, was deleted in the entomopathogenic fungus *Metarhizium robertsii.* It was found that MrHex1 was responsible for WB formation in *M. robertsii* and involved in sealing septal pores to maintain cell integrity and heterogeneity. Different assays indicated that, relative to the wild-type (WT) strain, ∆*Mrhex1* demonstrated a growth defect on a solid medium and substantial reductions of conidiation, appressorium formation and topical infectivity against insect hosts. However, there was no obvious virulence difference between WT and mutants during injection of insects. We also found that ∆*MrHex1* could tolerate different stress conditions like the WT and the gene-rescued mutant of *M. robertsii*, which is in contrast to the reports of the stress-response defects of the *Hex1* null mutants of other fungal species. In addition to revealing the phenotypic/functional alterations of the *Hex1* deletion mutants between different pathotype fungi, the results of this study may benefit the understanding of the evolution and WB-control of fungal entomopathogenicity.

## 1. Introduction

The filamentous ascomycete *Metarhizium robertsii* is an omnipresent and soil-dwelling pathogen of insects, ticks and mites [1,2]. It has been developed as a promising mycoinsecticide to control different insect pests and investigated as a genetically tractable system for studying fungus–insect interactions [3,4,5]. Similar to plant pathogens like the rice blast fungus *Magnaporthe oryzae*, *M. robertsii* infects insect hosts by penetrating host cuticles through the differentiation of the infection structure appressoria and build-up of the turgor pressure within appressorial cells [6,7]. The generation of the turgor pressure requires the accumulation of high concentrations of glycerol and or solutes within appressorial cells [8,9,10], which are separated from conidial mother cells by the formation of septa with the central septum pores sealed [11,12]. The mechanism of septal pore sealing in insect pathogens like *M. robertsii* is still unclear.

Fungal hyphal cells are separated by perforate septa, and filamentous fungi evolved with finely tuned strategies to balance the inter-cellular exchanges and the need for compartmentalization [13]. For ascomycete fungi belonging to the Pezizomycotina subphylum, Woronin bodies (WBs) are formed for plugging/unplugging the septal pores to regulate organelle exchanges between compartments, maintain hyphal cell heterogeneity and prevent excessive cytoplasmic bleeding in the event of hyphal damage [13,14,15]. WB is a peroxisome-type and hexagonal crystal-like organelle, which is membrane-bound and contains a dense core developed from the self-assembly of a single protein Hex-1, which has been first characterized in the model fungus *Neurospora crassa* in a very close association with septa [16,17]. The Hex-1-like proteins (either called HexA or Hex1) have then been identified and characterized in a few fungal species such as *Aspergillus oryzae* [15], *A. fumigatus* [18] and the plant pathogenic fungi like *M. oryzae* [12] and *Fusarium graminearum* [19]. The deletion of the *Hex1* gene resulted in the disappearance of WBs in fungal hyphae and the null mutants demonstrated impaired stress resistance abilities against the osmotic and cell-wall integrity interference agents, a dramatically reduced ability to survive wounding and or a reduced capacity in the infection of hosts [18,19]. For example, the *Hex-1* null mutant of *N. crassa* had reduced growth on a minimal medium and was impaired in sporulation [20]. After the deletion of *Aohex1* in *A. oryzae*, septal plugging was abolished and hyphal heterogeneity also affected [15]. The HexA of *A. fumigatus* was verified to be important for stress resistance and virulence [18]. For plant pathogenic fungi, it has been revealed that the formation of the Hex1-associated WB was required in *M. oryzae* for the development and function of the infection structures appressoria and therefore host colonization [12]. The disruption of the *Hex1* gene in *F. graminearum* reduced fungal asexual reproduction, infectivity and virus RNA accumulation in the infected cells when compared with the wild-type strain [19]. Likewise, the homologous *Hex1* gene was found to be required for WB formation, conidiation and the formation of the capturing trap in the nematophagous fungus *Arthrobotrys oligospora* [21]. The gene(s) responsible for WB formation and function in ascomycete entomopathogenic fungi has yet to be investigated.

In this study, it is intriguing to find the substantial length variation amongst the Hex1-domain-containing proteins from different fungi. We then performed the loss-of-function investigation of a homologous *Hex1* gene (MAA_00782, designated as *Mrhex1*) in the insect pathogenic fungus *M. robertsii*. It was found that *Mrhex1* was required in *M. robertsii* for WB formation, asexual growth and sporulation, appressorium differentiation and the topical infection of insect hosts. In contrast to the findings in other fungi, however, the null mutant of *MrHex1* could tolerate different stress conditions like the wild-type strain.

## 2. Materials and Methods

### 2.1. Strains and Culture Conditions

The wild-type (WT) strain and mutants of *M. robertsii* strain ARSEF 2575 were routinely cultured on potato dextrose agar (PDA; BD Difco, Franklin Lakes, USA) at 25 °C. Spore germination and appressorium induction assays were conducted using locust (*Locusta migratoria manilensis*) hind wings or the minimal medium (MM) (NaNO_3_, 6 g/L; KCl, 0.52 g/L; MgSO_4_·7H_2_O, 0.52 g/L; KH_2_PO_4_, 0.25 g/L) amended with 1% glycerol as the sole carbon resource (MM-Gly) [22]. For genomic DNA, RNA extractions and hyphae staining, fungal spores were cultured in Sabouraud dextrose broth (SDB; BD Difco, Franklin Lakes, USA) for three days at 25 °C and incubated at 200 rpm in a rotary shaker.

### 2.2. Protein Feature Characterization and Phylogenetic Analysis

Homologous Hex1 proteins were retrieved from GenBank for those containing the conserved S1_Hex1 domain (Table S1). The conservation analysis of the S1_Hex1 domains of 460 proteins obtained from different fungal species/strains was characterized with the program WebLogo (ver. 2.8.2) [23]. For phylogenetic analysis, 21 proteins selected from representative fungal species were aligned with the program Clustal X ver. 2.0 [24], and a bootstrapped (1000 replicates) neighbor-joining (NJ) tree was constructed with the program MEGA X [25] using the pairwise deletion of the alignment gaps and a Dayhoff substitution model.

### 2.3. Gene Deletion and Complementation

To determine the function of *MrHex1*, targeted deletion was performed by homologous recombination via the *Agrobacterium*-mediated transformation of the WT strain of *M. robertsii* as described before [26]. In brief, the 5′- and 3′- flanking sequences were amplified using the genomic DNA as a template with the primer pairs hex1UF (CGGAATTCGTACGGACCGATAAAACGTG) and hex1UR (CGGAATTCGAATGTCCTCCTTGATGTC), hex1DF (GCTCTAGACTGTCGACTGC-TTTCGAGTC) and hex1DR (GCTCTAGATAAGACACCCCATGTCAGC), respectively. The products were digested with the restriction enzymes *EcoR*I and *Xba*I, and then inserted into the same enzyme-treated binary vector pDHt-bar (conferring resistance against ammonium glufosinate) to produce the plasmid pBarhex1-KO for fungal transformation. For null mutant complementation, the full open reading frame (ORF) of the *Mrhex1* gene was amplified together with its promoter and terminator regions using the primer pairs hex1U (GGACTAGTGCACAGAGGACAAAACATGG) and hex1L (GGACTAGTTTACAGGCGAGAGCCGTGAA). The product was digested with *Spe*I, and then inserted into the binary vector pDHt-ben to produce the plasmid pBenhex1 (conferring resistance against benomyl) [27]. The drug-resistant mutants were isolated and verified by PCR and reverse transcription-PCR (RT-PCR) analyses with the primers hex1F (CACCACCACCATGACCAC) and hex1R (GAGAGCCGTGAATGACCTT). The β-tubulin gene (MAA_02081) was used as the control and amplified using the primers TubF and TubR [28].

### 2.4. Phenotyping, Cell Integrity and Stress Response Assays

To determine the effect of *MrHex1* deletion on fungal growth and conidiation, fungal cultures were grown on PDA and the colony diameters were measured at different times post inoculation. After growth for 18 days, conidial production was assayed and compared between the WT and mutants by two-tailed Student’s *t*-tests [29]. To determine cell integrity after gene deletion, the level of cellular content leakage was determined via the detection of free amino acids in liquid culture filtrates by reaction with ninhydrin [30]. Thus, the spores of the WT and mutants were collected from 14 day-old PDA plates and inoculated in SDB at 25 °C and 200 rpm for 4 days. Fungal mycelia were collected by filtration, washed twice with sterile distilled water and transferred to MM–N (i.e., without the addition of NaNO_3_ in MM) liquid medium for 24 h. The supernatants were collected by filtration and transferred (4 mL each) into the test tubes followed by the individual addition of 1 mL of 2% (*w*/*v*) of ninhydrin reagent and 1 mL phosphate buffer (pH, 8.0). The samples were mixed by vortexing prior to being treated in a boiling water bath for 15 min. After cooling at room temperature, the absorbance of each sample was recorded at 570 nm (A570) using a Biophotometer (Eppendorf). The reaction solutions were also transferred into 1.5 mL centrifuge tubes for photographing. The corresponding mycelia of each strain were dried in an oven at 50 °C overnight and weighed. The unit of A570 was then normalized with the mycelium dry weight of each sample. There were three replicates for each strain and the experiments were repeated twice. The two-tailed Student’s *t*-tests were conducted to compare the differences between strains.

For stress challenges, fungi were grown on PDA or PDA amended with the final concentrations of 0.01% sodium dodecyl sulphate (SDS), 200 μg/mL Calcofluor white and 250 μg/mL Congo red for cell wall integrity challenges; 50 μM farnesol for antifungal resistance, and 1.5 M KCl and 1 M Sorbitol for osmotic challenges [18,26], respectively. For inoculation, 2 μL of the 10-fold diluted spore suspensions (2 × 10^7^ conidia/mL) were spotted onto the plates and incubated at 25 °C for three days.

### 2.5. Microscopy Observations

To determine the effect of *MrHex1* deletion on WB formation, a transmission electron microscope (TEM) analysis was conducted as described before [27]. The spores of the WT and mutants were inoculated in SDB for three days and the mycelia were harvested by filtration. After washing twice with distilled water, fungal samples were fixed in 2.5% glutaraldehyde in 0.1 M phosphate buffer solution (PBS; pH, 7.2) at 4 °C for 12 h, rinsed three times in the phosphate buffer, and fixed overnight in 1% osmium tetroxide buffered in 0.1 M cacodylate (pH, 7.0) at 4 °C. After rinsing three times with the phosphate buffer, samples were dehydrated in an ethanol gradients, infiltrated with a gradient series of epoxy propane, and then embedded in Epon resin for sectioning [27]. The ultrathin samples were treated in 2% uranium acetate and then lead citrate prior to the observations under a TEM (H-7650; Hitachi).

The mycelia collected from SDB were also used for fluorescent staining. After washing with PBS, the mycelia of each strain were jointly stained with DAPI (4′6-diamidino-2-phenylindole, Sigma-Aldrich, St. Louis, USA) and Calcofluor white (CW, Sigma-Aldrich) to detect nuclei and cell septa, respectively. A stock solution of DAPI (100 μg/mL) was prepared in water and diluted to 1–2 μg/mL in PBS for staining for 30 min. After washing with PBS three times, the samples were then treated with CW solution (4 μg/mL) buffered in 10% potassium hydroxide for 1 min prior to the observations with an Olympus microscope (BX51-33P, Tokyo, Japan).

### 2.6. Appressorium Induction and Insect Bioassays

Appressorium formation of the WT and mutants were induced on both a hydrophobic surface and locust hind wings [28]. Briefly, the spores of each strain were inoculated into individual polystyrene petri dishes (6 cm in diameter) containing 2 mL MM-Gly at a final concentration of 2 × 10^5^ conidia/mL. After incubation for 24 h, the appressorium differentiation rates were recorded for > 300 conidia under a microscope. The locust hind wings were surface sterilized in 37% H_2_O_2_ for 5 min, washed twice with sterile water and immersed in conidial suspensions (2 × 10^7^ spores/mL) for 20 s. The inoculated wings were lined on 0.8% water agar at 25 °C for 16 h. The Student’s *t*-tests were conducted to compare the differences between strains.

Insect bioassays for the WT and mutants were conducted using the newly emerged last instar larvae of the mealworm *Tenebrio molitor* and silkworm *Bombyx mori*. Conidia were harvested from the two-week old PDA plates and suspended in 0.05% Tween-20 at the concentration of 1 × 10^7^ conidia/mL. Insects were chilled on ice before immersion in spore suspensions for 30 s. In addition, injection assays were performed using the silkworm larvae. Each insect was injected at the second proleg with 10 µL of the suspensions each containing 1 × 10^6^ conidia/mL. The mortality was recorded every 12 h and the median lethal time (LT_50_) was calculated by Kaplan–Meier analysis [31]. The control insects were treated with 0.05% Tween-20. Each treatment had three replicates with 15 insects each and the experiments were repeated twice.

## 3. Results and Discussions

### 3.1. Length Variation of the Hex1 Proteins with Conserved C-termini

**Figure 1:**
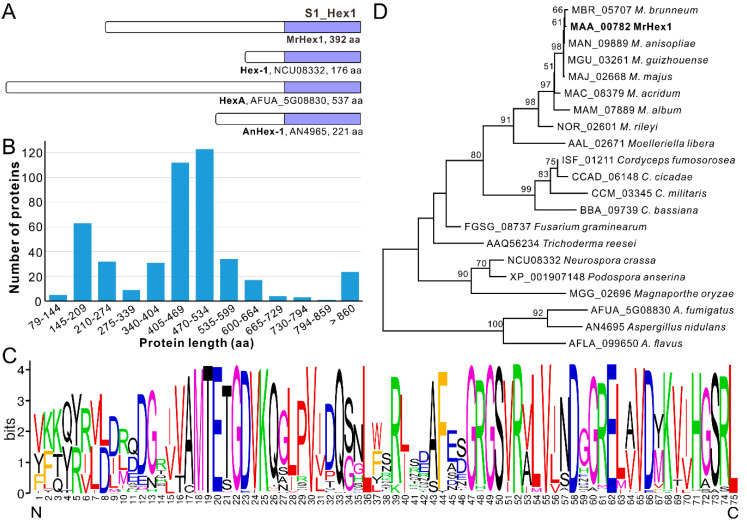
Schematic structuring and phylogenetic analysis of the selected Hex1 proteins. (**A**) Schematic structuring of MrHex1 and the selected homologues. Selected proteins are: Hex-1 from *Neurospora crassa*, HexA from *Aspergillus fumigatus* and AnHex-1 from *A. nidulans.* (**B**) Length variations of the Hex1 proteins from different fungi. *X* axis represents the amino acid (aa) length region of proteins. *Y* axis represents the number of proteins belonging to different length regions. (**C**) Conservation analysis of the Hex1-domain sequences. The sequences (75 aa each) were extracted from 460 Hex1 proteins from different fungal species. The N and C letters labeled at the bottom represent the N- and C-termini of the Hex1 domains. (**D**) Phylogenetic analysis of the selected Hex1 proteins. Protein sequences were retrieved from the selected fungal species and aligned to generate a neighbor joining tree with a Dayhoff substitution model and 1000 bootstrap replicates.

The single copy and complete ORF of *Mrhex1* (MAA_00782) encodes a protein possessing 392 amino acid (aa) residues and containing a carboxyl-terminal S1_Hex1 domain (75 aa) like other proteins such as Hex-1 of *N. crassa* and HexA of *A. fumigatus* [18,20], however, with substantial total length variations between each other (Figure 1A). Further survey of the S1_Hex1 domain proteins catalogued in GenBank obtained 460 proteins (single copy within each genome) from those fungal species belonging to the clade Sordaromyceta of the subphylum Pezizomycotina (Ascomycota) (Table S1). Unexpectedly, the substantial length variation was further evident for the Hex1 proteins from different fungal species, ranging from 79 aa (EPQ66756, *Blumeria graminis f. sp. tritici*) to 2958 aa (ERF74742, *Endocarpon pusillum*) (Table S1). The misannotation of some of these proteins could not be precluded. Statistically, the major distribution of Hex1 protein length is within the regions 470–534 aa (26.7%, 123/460), 405–469 aa (24.3%, 112/460) and 145–209 aa (13.9%, 64/460) (Figure 1B). The last group includes those characterized in *N. crassa* (Hex-1, NCU08332, 176 aa) and *A. nidulans* (AnHex1, AN4965, 221 aa). Length variations were also evident in different species from the same genus. For example, the Hex1 homologues from *Metarhizium* genus vary from 392 aa (MAA_00782 and MAN_09889, *M. anisopliae*) to 423 aa (MAC_08379, *M. acridum*) and 454 aa (NOR_02601, *M. rileyi*). Likewise, the proteins from the *Aspergillus* and other fungal genera are also highly variable in total length (Table S1). Similar to this finding, length differences have also been observed between other proteins belonging to the same family. Some protein domains are functionally permissive to length variation (termed length-deviant domains) while some others are less tolerant to length alteration (termed length-rigid domains) [32]. Considering the conserved function of Hex1 in WB formation in different fungi [13], it is therefore length-deviant for Hex1 proteins in term of their full lengths. It was found that the Hex-1 cleavage occurred in *N. crassa* [20]. The mature and functional length of Hex1 proteins remains to be determined in different fungi.

Irrespective of clear length variations among Hex1 proteins, a highly conserved C-terminus S1_Hex1 domain with 75 aa residues is evident in each Hex1 protein, a typical feature of the length-rigid domain (Figure 1A; Table S1). In particular, the characteristic and specific peroxisome-targeting signal 1 (PTS1) tripeptide S/A-R/S-L [17] is present at the C-terminal of MrHex1 and other proteins (Figure 1C; Table S1), which is different from the consensus PTS1 motif S/A/C-K/R/H-L/M reported before [13,17]. In particular, the PTS1 motif A-S-L is found from the putative Hex1 proteins of the plant pathogen *Monosporascus* genus and an S-S-L pattern from the Hex1 proteins of the *Valsa* genus (Table S1), where the second residue of serine (S) has not been suspected before. A phylogenetic NJ tree generated with 21 selected Hex1 proteins revealed that the clustering pattern of these proteins largely correlated with fungal speciation relationships (Figure 1D). For example, consistent with previous analyses [33,34], the Hex1 proteins from *Metarhizium* species evolved following the trajectory from the specialists (*M. rileyi* and *M. album*) to the generalist species (e.g., *M. robertsii* and *M. brunneum*) with a broad host range. In this respect, *Hex1* might have evolved by following fungal divergence and speciation after its birth in the ancestor of the Pezizomycotina fungi.

### 3.2. MrHex1 Effecting on Fungal Growth, Sporulation and Stress Responses

**Figure 2:**
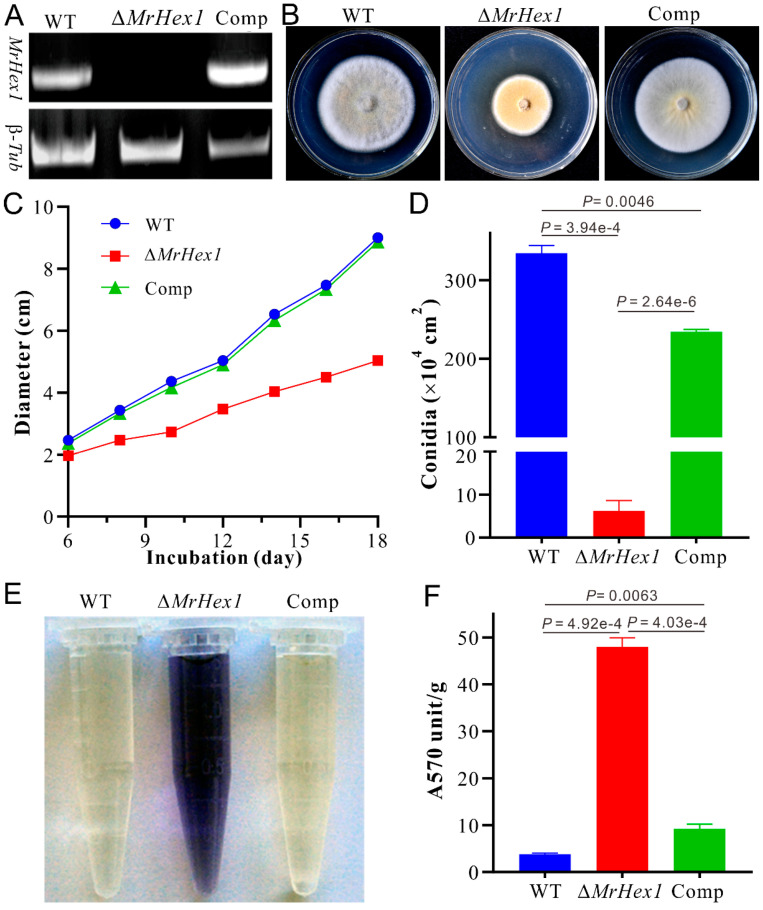
Gene deletion and phenotypic characterizations: (**A**) RT-PCR verification of gene deletion and complementation. Comp, the complemented mutant; β-*Tub*, the β-tubulin gene used as a control. (**B**) Phenotypic characterization of the wild-type (WT) and mutants after growth on potato dextrose agar (PDA) for 14 days. (**C**) Time-scale growth assays by measuring colony diameters. (**D**) The quantification of conidial production by WT and mutants after growth on PDA for 18 days. (**E**) Culture filtrates of different strains after reaction with ninhydrin. (**F**) Photometric estimation of the leaked amino acids after reaction with ninhydrin. The unit absorbance of A570 was normalized to the mycelium dry weight. Error bar on top of each column represents standard deviation.

**Figure 3:**
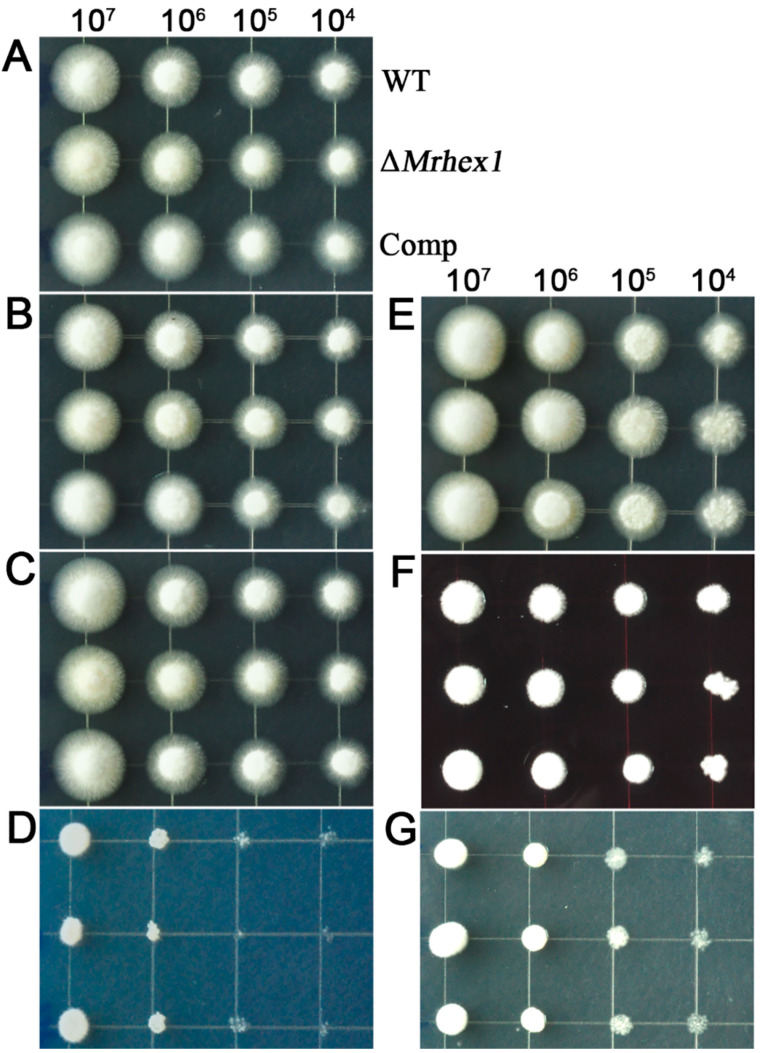
Stress response assays. The spores of the WT and mutants were inoculated on PDA (**A**), PDA amended with farnesol at 50 μM (**B**), PDA plus Calcofluor white at 200 μg/mL (**C**), PDA plus KCl at 1.5 M (**D**), PDA plus sodium dodecyl sulphate (SDS) at 0.01% (**E**)**,** PDA plus Congo red at 250 μg/mL (**F**) and PDA plus sorbitol at 1 M (**G**). The phenotypes were photographed after inoculation with 2 μL of spore suspensions (started at 2 × 10^7^ conidia/mL) diluted 10-fold for three days.

By checking the previous RNA-seq transcriptome data, relative to the conidial sample, *MrHex1* was found to be highly transcribed by the fungus during the formation of appressoria on locust wings [35]. To determine the function of *MrHex1* in *M. robertsii*, the gene was deleted and the obtained null mutant was also complemented by the verification of RT-PCR analysis (Figure 2A). Phenotypic growth assays showed that the deletion of *MrHex1* substantially reduced the fungal growth rate when compared with the WT and complemented (Comp) strains (Figure 2B,C). In addition, we found that the sporulation ability of ∆*MrHex1* was severely (*P* = 3.94 × 10^−4^) impaired when compared with that of the WT (Figure 2D). Unexpectedly, the gene-rescued mutant Comp also had a reduced level of conidiation when compared with the WT (*P* = 2.64 × 10^−6^). Otherwise, relative to the WT, both the null and rescued mutants did not show obvious defects in their stress responses against the challenges with the detergent SDS, osmotic stressors KCl and sorbitol, antifungal agent farnesol or cell wall biosynthesis inhibitors CW and Congo red (Figure 3).

The requirement of Hex1 for asexual growth and sporulation has also been found in a few fungal species like *N. crassa* [17], *A. oligospora* [21], *F. graminearum* [19] and *M. oryzae* [12]. However, in contrast, the ∆*HexA* of *A. fumigutas* showed normal growth and sporulation like the WT strain [18]. Thus, similar to the observation of functional divergence between the conserved transcription factors in different fungi [36,37], Hex1 also shows functional alterations in different fungi. The fact that no obvious differences were observed in the stress responses between WT and Δ*MrHex1* provided further supports of species-dependent functional variations of Hex1 in different fungi. For example, it has been found that, in contrast to ∆*MrHex1* and ∆*HexA* of *A. fumigatus* [18], *Hex1* null mutant of *A. oligospora* was sensitive to osmotic stress [21]. However, relative to the WT of *A. fumigatus*, Δ*HexA* became sensitive to SDS, farnesol, CW and Congo red [18], which was not the case for Δ*MrHex1* as we showed.

### 3.3. Requirement of Mrhex1 for Woronin Body Formation and Maintaining Cell Integrity

**Figure 4:**
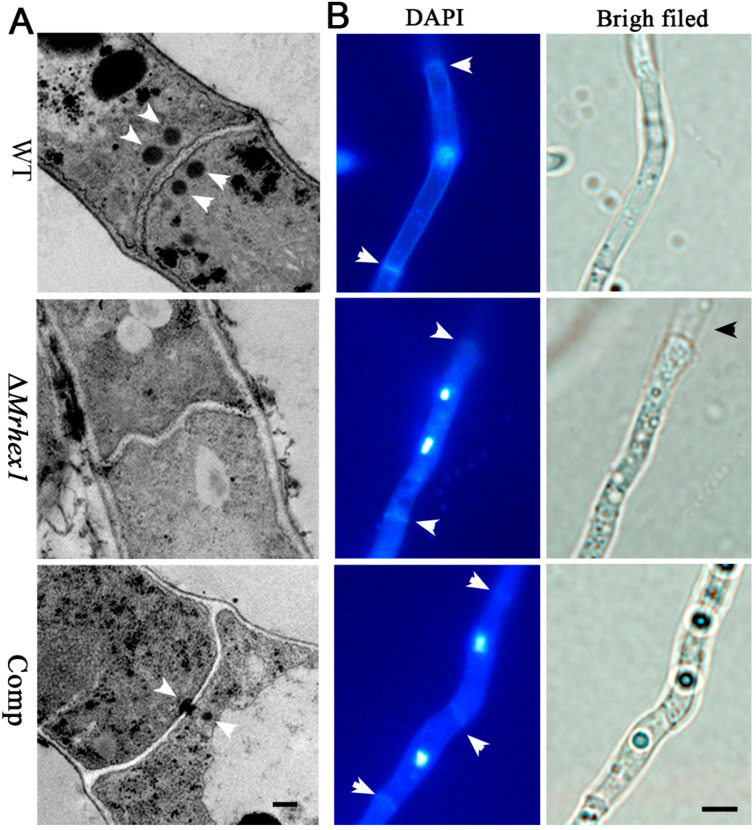
Microscopic observations: (**A**) transmission electron microscope observation showing the presence or absence of Woronin bodies (arrowed) in the WT and mutant cells. Bar, 0.5 μm; (**B**) the co-staining of the mycelium cells for detecting nuclei and septa (arrowed). The broken end of the Δ*MrHex1* mycelium is arrowed for its bright field image. Bar, 5 μm.

Hex1 is the major WB protein in Pezizomycontina fungi [13,16,20]. To determine the function of MrHex1 in WB formation in *M. robertsii*, mycelial samples of the WT and mutant strains were subject to TEM analysis. As a result, the dense and characteristic WBs were evident on both sides of the WT cell septa but absent in Δ*MrHex1*. For Comp, after the examination of multiple section samples, the WT-like distribution of WBs was not observed but the WBs were found to be plugged or anchored in proximity to the septum pore (Figure 4A). Thus, MrHex1 is similarly required for WB formation in *M. robertsii*. This kind of WB number and positioning differences between WT and the complemented mutant has also been found in *F. graminearum* [19] and *A. oligospora* [21]. It is noteworthy that WB positioning and localization are associated with the WB enveloping protein (i.e., the Woronin sorting complex protein, WSC) and a tethering protein Leashin (Lah) [38]. The *N. crassa* WSC-like protein (NCU07842 vs. MAA_02499, 71% identity at amino acid level) is present in *M. robertsii*. However, in contrast to the finding in *Aspergillus* fungi [39], the large and nonconserved Lah-like protein remains elusive in *M. robertsii*. In addition, it has been known that the proper function of some genes requires their positions preferentially located in genomes [40]. The importance of the *Hex1* gene positioning remains to be determined for function. It could not be precluded at this stage that the imperfect issue of gene rescue might be due to the non-original position insertion.

The anchoring of WBs to the septum pore in fungal cells is essential for preventing cytoplasmic leakage after cell damage [11], and maintaining cell integrity and heterogeneity [13]. We first performed ninhydrin reaction assays to determine if any difference between WT and mutants in terms of the amino acid leakage in culture filtrates. The results indicated that a deep purple color, the result of amino acid reaction with ninhydrin, was evident for the ∆*MrHex1* sample but not for the WT and Comp strains (Figure 2E). Consistently, the photometric assays indicated that the A570 value of the ∆*MrHex1* sample was significantly higher than those of the WT (*P* = 4.92 × 10^−4^) and Comp (*P* = 4.03 × 10^−4^) (Figure 2F). It was also found that the A570 value of Comp was higher than that of the WT (*P* = 0.0063) for an unclear reason. We also performed the joint fluorescent staining of different strains for detecting the distribution pattern of the nuclei within each hyphal cell. The results showed that only one nucleus was observed within one hyphal cell of the WT and Comp whereas more than one nucleus were frequently evident in Δ*Mrhex1* cells, especially within the cells close to the injured end (Figure 4B). MrHex1 is therefore functionally important in maintaining cell integrity and heterogeneity in *M. robertsii*. Likewise, it has been shown that the hyphal heterogeneity of *A. oryzae Hex1* null mutant was affected [15]. It has also been shown that the peroxisome-related WB formation affects fungal secondary metabolisms [13], which remains to be determined in *M. robertsii*.

### 3.4. Defects of Mrhex1 Null Mutant in Appressorium Formation and Topical Infection of Insects

**Figure 5:**
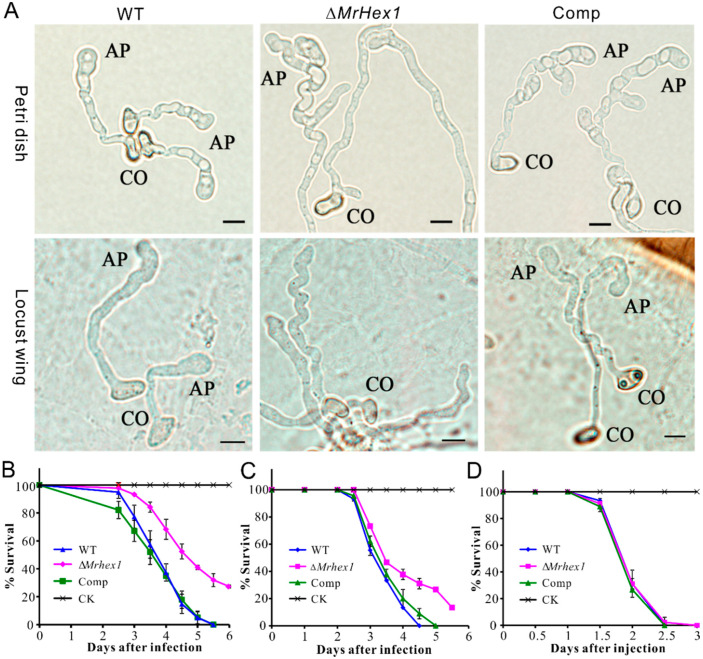
Appressorium induction and insect survival assays. (**A**) Microscopic examination of appressorium formation by the WT and mutants on hydrophobic surface (upper panels) and locust hind wings (lower panels). CO, conidium; AP, appressorium. Bar, 5 μm. (**B**) Survival of the mealworm larvae after topical infection. (**C**) Survival of the silkworm larvae after topical infection. (**D**) Survival of the silkworm larvae after injection.

We then performed infection structure induction and insect bioassays with the WT and mutant strains. Appressorium formation was induced on both the hydrophobic surfaces and locust hind wings. As a result, we found that appressorium production was considerably impaired for Δ*MrHex1* when compared with the WT and Comp under both conditions (Figure 5A). Statistically, the rate of appressorium production by Δ*Mrhex1* (23.3% ± 2.53) declined significantly (*P* < 0.001) when compared with those formed by WT (83.6% ± 5.69) and Comp (82.9% ± 4.38) on a hydrophobic surface. The failure of septal pore sealing might lead to the defects in building up turgor pressure within appressorium cells. Considering that the mutants of *M. robertsii* with impaired abilities in generating cellular turgor pressure could still form appressoria [10,27,41], the defect of ∆*MrHex1* in appressorium formation might not be due to the turgor generation failure of the mutant. The exact mechanism between WB and infection structure formations requires further investigation.

Consistent with the mutant defect in appressorium formation, the topical infection of the mealworm and silkworm larvae revealed that the virulence reduction of ∆*MrHex1* was evident (Figure 5B,C). Thus, the LT_50_ value of Δ*MrHex1* (4.98 ± 0.18 days) was significantly higher than those of the WT (3.94 ± 0.12 days; χ^2^ = 25.12, *P* < 0.0001) and Comp (3.80 ± 0.15 days; χ^2^ = 22.24, *P* < 0.0001) during the topical infection of *T. molitor* larvae. For the topical infection of silkworm larvae, the LT_50_ value of Δ*MrHex1* (4.02 ± 0.14 days) was also higher than those of the WT (3.48 ± 0.09 days; χ^2^ = 11.04, *P* < 0.001) and Comp (3.62 ± 0.10 days; χ^2^ = 7.0, *P* < 0.01). However, survival dynamics were similar between the WT and mutant strains during the injection assays (χ^2^ < 2.0, *P* > 0.1) of the silkworm larvae (Figure 5D). These results confirmed that the deletion of *MrHex1* impaired the fungal ability to penetrate host cuticles due to the null mutant defect in appressorium formation and or the generation of turgor pressure. Considering that the sporulation ability of Δ*MrHex1* was impaired, the mycosis of insect cadavers killed by either topical infection or injection might also be negatively affected for Δ*MrHex1* when compared with the WT and Comp strains.

Similar to our observations, the defects in appressorium formation and therefore virulence reduction were also observed in the ∆*Hex1* of *M. oryzae* [12]. Likewise, the failure of trap formation was evident for the ∆*AoHex1* of *A. oligospora* and the mutant lost its ability to capture nematodes [21]. Both the deletion and overexpression of *FgHex1* in *F. graminearum* reduced fungal infectivity [19]. However, intriguingly, the deletion of *CoHex1* in the cucumber anthracnose fungus *Colletotrichum orbiculare* did not produce any detectable defects in appressorium formation and infectivity [42]. This kind of species-dependent phenotypic diversity of *Hex1* deletion mutants indicates again the functional alterations of this conserved gene in different fungi.

## 4. Conclusions

In this study, the WB-formation protein MrHex1 was characterized in the insect pathogenic fungus *M. robertsii*. Unexpectedly, we first found the substantial length variation among Hex1 proteins from different fungi but each with a highly conserved C-terminal tail and the characteristic PTS1 sorting signature. Taken together with the finding that MrHex1 is similarly required for WB formation in *M. robertsii*, the data suggest that the length variation of Hex1 proteins might have no hindrance for their similar functions in WB formation in different fungi. However, phenotypic alterations were clearly evident between ∆*MrHex1* and *Hex1* null mutants of other fungi. In particular, unlike other fungal mutants [18,19], ∆*Mrhex1* demonstrated an equal tolerance to different stress conditions like the WT and Comp of *M. robertsii*. This kind of phenotypic and functional divergence of *Hex1* genes implies the necessity of investigating evolutionarily conserved genes in different fungal pathotypes. The finding that MrHex1 is required in *M. robertsii* for infection structure formation and the topical infection of insect hosts advances our understanding of the control and evolution of fungal entomopathogenicity. Future efforts are still required to investigate the mature type of Hex1 within fungal cells, the relationship between gene positioning and function, and the feasibility of functional complementation of the length-varied Hex1 among different fungi.

## Supplementary Materials

The following are available online at https://www.mdpi.com/2309-608X/6/3/172/s1, Table S1: Characteristics of the Hex1 proteins retrieved from different fungal species.
